# STAKEHOLDER PRIORITIES FOR REHABILITATION TO SUPPORT FUNCTIONING AT HOME AND WORK AMONG PEOPLE ON SICK LEAVE DUE TO LOW BACK PAIN: A GROUP CONCEPT MAPPING STUDY

**DOI:** 10.2340/jrm.v58.46346

**Published:** 2026-11-13

**Authors:** Katrine Schilling ANDERSEN, Eva Ejlersen WÆHRENS, Claus Vinther NIELSEN, Reuben ESCORPIZO, Bodil Kaalund BRUUN, Marc Sampedro PILEGAARD

**Affiliations:** 1 Department of Social Medicine and Rehabilitation, Gødstrup Hospital, Central Denmark Region, Denmark; 2 Department of Public Health, Aarhus University, Aarhus, Denmark; 3 Occupation-Centred Occupational Therapy, Parker Institute, Copenhagen University Hospital – Bispebjerg and Frederiksberg, Copenhagen, Denmark; 4 DEFACTUM, Central Denmark Region, Aarhus, Denmark; 5 User Perspectives and Community-based Interventions, the Research Group for Occupational Science, Department of Public Health, University of Southern Denmark, Odense, Denmark; 6 Department of Rehabilitation and Movement Science, The University of Vermont, Burlington, VT, USA

**Keywords:** activities of daily living, concept mapping, low back pain, rehabilitation, return to work, sick leave, stakeholder participation

## Abstract

**Objective:**

To identify, organize, and prioritize stakeholder-defined components of rehabilitation to support functioning in everyday life, at home and at work, among people on sick leave due to low back pain.

**Design:**

Participatory mixed-methods study using group concept mapping conducted in Denmark.

**Subjects/patients:**

Participants with lived experience of low back pain and sickness absence, healthcare and employment professionals, workplace representatives, and researchers.

**Methods:**

Stakeholders completed online brainstorming, sorting, and rating, followed by a validation workshop. Multidimensional scaling and cluster analysis were applied.

**Results:**

Brainstorming involved 101 participants and generated 190 ideas, synthesized into 125 statements. Sorting and rating data were provided by 23 and 36 participants, respectively. Eleven participants attended the validation workshop. Six clusters consisted of self-management, physical training and activity, workplace involvement, interdisciplinary coordination, individual support, and extended assessment. Thirty-nine statements (31.2%) were rated very important and all clusters received high importance ratings.

**Conclusion:**

Stakeholder priorities encompassed physical, psychological, social, and work-related aspects of rehabilitation, consistent with a biopsychosocial perspective. Rehabilitation was conceptualized as a coordinated, adaptive, and person-centred process across sectors and everyday-life contexts. Although represented across clusters, functioning at home did not emerge as a distinct domain and requires further attention in low back pain rehabilitation.

## LAY ABSTRACT

People on sick leave because of low back pain may experience difficulties functioning at home and at work. Developing rehabilitation requires research evidence and perspectives from lived experience and practice.

Through group concept mapping, we asked people with lived experience of low back pain, professionals, workplace representatives, and researchers which types of support they considered important. They identified 6 areas: *support for self-management*, *physical training and movement*, *support at work*, *coordination between services, individual support*, and *extra assessment and treatment support*. Stakeholders suggested rehabilitation as a coordinated and flexible process, addressing aspects of a person’s life. Functioning at home was represented across several areas but not as a separate area of support. The findings can inform the development of rehabilitation support for people on sick leave due to low back pain. However, they do not show which types of support are effective or how to combine or deliver the support.

Low back pain (LBP) is the leading cause of disability worldwide (1). It is associated with substantial socioeconomic costs through healthcare utilization, long-term sick leave, reduced productivity, and early exit from the labour market (2, 3). Although many episodes of LBP resolve, recurrence is common, and a substantial proportion of individuals experience persistent pain and long-term disability (4). Because the clinical course of LBP varies considerably, it is commonly categorized according to symptom duration as acute (< 6 weeks), subacute (6 to < 12 weeks), or persistent/chronic (≥ 12 weeks) (5).

The persistence of LBP reflects a complex interplay of biological, psychological, social, and contextual factors (4, 6, 7). These interacting factors contribute not only to pain, but also to impairments in body functions and structures, limitations in activities, and restrictions in participation, as conceptualized by the International Classification of Functioning, Disability and Health (ICF) (8). Among individuals on sick leave due to subacute or persistent LBP, these limitations often affect management of everyday activities at home, participation at work and other social roles, highlighting functioning as a multidimensional construct (9–11).

To address this complexity, multidisciplinary biopsychosocial rehabilitation is recommended for individuals with subacute and persistent LBP (4, 12, 13). However, evidence shows moderate and inconsistent effects on pain, disability, and work-related outcomes (12–14). A key challenge in the literature is the insufficient specification of intervention content, which limits the understanding of which components are essential for improving functioning (12–15). This lack of conceptual clarity is particularly evident in workplace-integrated biopsychosocial rehabilitation (16). Although workplace interventions may improve work-related outcomes, they remain largely focused on physical components, with limited integration of psychological and social dimensions (16–18). This narrow operationalization of the biopsychosocial model may fail to adequately address the broader dimensions of functioning (9, 19–22). These findings indicate that biopsychosocial interventions addressing both everyday functioning outside work and work participation are not yet clearly defined (12–15, 19).

Developing complex rehabilitation interventions requires the integration of research evidence and theory with clinical expertise, practice-based knowledge, and lived experience (23, 24). While existing research can indicate which approaches have previously been evaluated, stakeholder perspectives can identify components, relationships, and contextual considerations that may be insufficiently represented in the literature. Accordingly, stakeholder-informed approaches are needed to clarify, conceptualize, and prioritize core components of rehabilitation for individuals on sick leave due to LBP. Group concept mapping (GCM) provides a structured, participatory methodology for identifying and conceptually organising stakeholder perspectives relevant to functioning in everyday life and work participation.

The aim of this study was to identify, organize, and prioritize stakeholder-defined key components of rehabilitation to support functioning in everyday life, both at home and at work, among people on sick leave due to LBP.

## METHODS

### Study design and methodological justification

GCM is a participatory mixed-method approach combining qualitative idea generation with quantitative structuring and multivariate analyses to identify, organize, and prioritize stakeholder perspectives systematically (25–27). The study followed Trochim’s 6 phases: preparation, idea generation, statement structuring, concept mapping analysis, interpretation, and utilization (27, 28).

Reporting followed the ConMapT guideline (28).

According to Danish regulations, ethical approval was not required. The study was registered in the Central Denmark Region (case no. 1-10-72-116-25). All participants provided informed consent, and data handling complied with the General Data Protection Regulation (GDPR). Participants received no financial or other compensation for taking part in the study.

### Study setting

The study was conducted within the Danish welfare system, where responsibility for rehabilitation and sickness-absence management is distributed across healthcare, municipal employment services, and workplaces. Denmark comprises 5 regions and 98 municipalities. General practitioners usually provide initial assessment and management of LBP and refer patients to specialized services when indicated. Regional hospitals provide specialized assessment and treatment, while municipalities provide community-based rehabilitation. Municipal employment services administer sickness-benefit follow-up and support labour-market participation, and employers support return to work through dialogue and workplace adjustments. Specialized social medicine and occupational medicine services may provide assessment and advice in complex or work-related cases, but Denmark has no universal occupational health service responsible for coordinating rehabilitation or return to work. Educational services are involved only when education or vocational retraining is relevant.

### Phase 1: Preparation

Two stakeholder advisory panels contributed to refining the study aim, the recruitment strategy, and the focus prompt – the statement stem used to elicit ideas. The citizen panel included people with lived experience of LBP and sickness absence (*n* = 7), while the professional panel consisted of representatives from municipal employment and healthcare services (*n* = 4), a regional department of social medicine (*n* = 1), and a specialized spine care unit (*n* = 1). Separate online and in-person workshops were held to facilitate dialogue and minimze power imbalances. The panels refined the focus prompt to ensure that it was understandable across stakeholder groups and encompassed functioning at home and at work.

*Determining the focus prompt.* The focus prompt was developed through an iterative, participatory process with the 2 stakeholder advisory panels, which contributed to refining the wording to ensure relevance across stakeholder groups. Emphasis was placed on making the focus prompt understandable and applicable to both everyday functioning and participation at work.

The final focus prompt was formulated as:

“For a person on sick leave due to low back pain to have the best possible everyday life, both at home and at work, in my experience, there is a need for …”

*Participants and recruitment.* A stakeholder analysis conducted with the stakeholder advisory panels identified 4 relevant groups: adults (≥ 18 years) with lived experience of LBP and sickness absence, healthcare and employment professionals, workplace representatives, and researchers. Participants were purposively recruited to ensure variation in perspectives and geographical representation across all 5 regions of Denmark. People with lived experience of LBP and sickness absence were recruited via social media posts in patient-oriented online public networks. Healthcare and employment professionals comprised an interdisciplinary group with experience in rehabilitation and/or case management across the healthcare and employment sectors. Recruitment targeted municipal primary healthcare services, employment services, general practice, and specialized regional hospital units for spine care, social medicine, and occupational and environmental medicine. Invitations were distributed to relevant staff via managers. Workplace representatives included employers, managers, human resources staff, and other workplace stakeholders with experience of supporting employees on sick leave due to musculoskeletal conditions. They were recruited through direct contact with workplaces across Denmark, with workplace management asked to forward invitations to relevant staff. Researchers and national experts with expertise in LBP, rehabilitation, and return to work were invited through national professional and research networks and relevant research units. All brainstorming participants were invited to subsequent phases, and participation remained voluntary throughout.

*Sample size considerations.* In GCM studies, sample size is guided by conceptual breadth and map stability rather than statistical power (27, 28). Recruitment therefore prioritized broad stakeholder representation in the idea-generation phase. Previous studies suggest that 40–100 participants are appropriate for idea generation, whereas smaller samples of 20–40 participants are sufficient for the structuring phases to produce stable cluster solutions (27, 28).

### Phase 2: Generating ideas

*Brainstorming.* Between 28 October and 21 November 2025 participants individually completed the anonymous online brainstorming task. Participants were asked to respond to the focus prompt by generating multiple brief responses, each expressing a single idea. The task was administered through the web-based Concept System® GroupWisdom™ platform (29), which was also used for the subsequent sorting and rating tasks and for data management and concept mapping analyses. Written instructions were provided within the platform, and participants experiencing difficulties were offered technical support by the research team.

*Statement reduction.* Two researchers independently reduced the brainstormed statements through a two-step process. First, duplicate statements were removed, multi-idea responses were split into separate statements, and minor linguistic revisions were made to improve clarity while preserving original meaning. Second, statements with substantial conceptual overlap were merged to create a manageable set for the structuring phase, with a maximum of 125 statements, consistent with established GCM procedures (27, 28). These steps were first conducted independently by the 2 researchers and then finalized through consensus, resulting in a set of unique statements representing stakeholder perspectives. Examples of the transformation from original responses to final statements are provided in Table SI.

### Phase 3: Structuring the statements

All participants from Phase 2 were invited to complete sorting and rating tasks using GroupWisdom™ (29). Participants individually sorted the statements into self-defined piles based on perceived conceptual similarity and assigned a label to each pile. Each statement was then rated on a 4-point Likert scale assessing importance (1 = not at all important, 4 = extremely important). Before analysis, the first author reviewed the exported task-completion data and assessed sorting and rating responses separately. Sorting responses were included if a participant sorted at least 75% of the statements into more than 1 pile and labelled each pile, as instructed. Rating responses were included if at least 75% of the statements were rated. Consequently, a participant could contribute to 1 analysis even if their response was excluded from the other.

### Phase 4: Concept mapping analysis

Background variables were summarized descriptively using frequencies and medians, as appropriate. In GroupWisdom™, sorting data were converted into a similarity matrix and analysed using non-metric multidimensional scaling to produce a 2-dimensional point map. Model fit was assessed using the stress value, with values between 0.20 and 0.36 considered acceptable (27). Ward’s hierarchical cluster analysis was conducted to identify cluster solutions. The number of clusters was not predetermined. Instead, cluster solutions were examined iteratively based on conceptual interpretability and the extent to which clusters represented distinct and meaningful domains. Bridging values were used to support interpretation, with lower values indicating greater conceptual coherence. Median importance ratings were calculated at statement and cluster levels and compared between participants with lived experience and professionals. Statements with a median rating of 4 were classified as very important.

### Phase 5: Interpreting the map

*Data interpretation process.* Participants who had completed sorting and/or rating were invited to a validation workshop to assess the proposed cluster solution. After receiving information regarding the point map, cluster solutions, and ratings, participants reviewed cluster meanings and boundaries, labels, and statement placements. Proposed modifications were considered according to conceptual fit, cluster coherence, and consistency with the overall map, with bridging values and rating patterns used as supporting information. No formal voting procedure or numerical threshold was used. For each proposed modification, the facilitator summarized the discussion and invited further comments or objections. Consensus was considered reached when participants agreed with the proposed modification and no substantive objections remained. All disagreements were resolved through continued discussion. Following the workshop, the research team reviewed the modifications against the stated interpretive criteria and incorporated all consensus-based recommendations into the final concept map.

### Phase 6: Utilization

The utilization phase involved translating the final validated concept map into an author-developed conceptual model to inform subsequent complex intervention development. The authors examined the relationships among the clusters and considered how established rehabilitation theories and frameworks could support their organization within a coherent rehabilitation process. Through iterative discussions, the clusters were interpreted according to their potential roles and relationships within such a process, while retaining their connection to the participant-validated concept map. The resulting model was intended as a conceptual synthesis rather than a fully specified intervention or programme theory.

## RESULTS

### Participants and flow through the group concept-mapping process

A total of 101 participants contributed to brainstorming, 23 provided valid sorting data, and 36 provided valid rating data (Fig. 1). Twenty-six sorting responses were excluded because fewer than 75% of statements had been sorted, 2 because pile labels were missing, and 8 rating responses because fewer than 75% of statements had been rated.

**Fig. 1:**
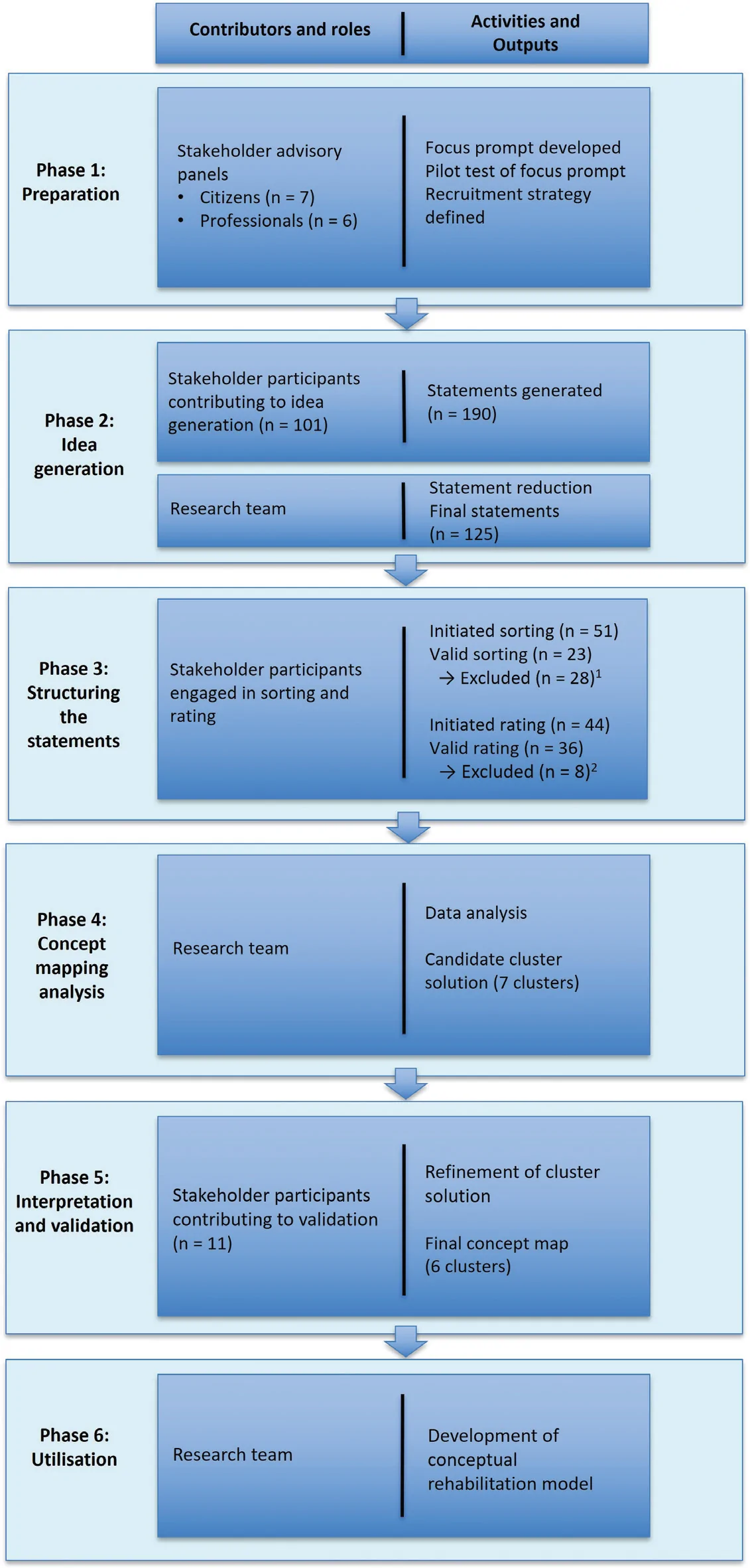
Flow through the group concept mapping process. The figure illustrates stakeholder advisory input, stakeholder participation, research team contributions, key activities, and outputs across the 6 phases of the group concept mapping process. ^1^Excluded sorting data comprised participants who sorted < 75% of the statements (*n* = 26) or had missing group labels (*n* = 2). ^2^Excluded rating data comprised participants who rated < 75% of the statements (*n* = 8).

### Participant characteristics

Table I presents participant characteristics. Participants across the GCM phases represented all predefined stakeholder groups, including people with lived experience of LBP, workplace representatives, researchers, and a broad range of healthcare and employment professionals. Participants represented all predefined stakeholder groups and all 5 Danish regions, although most were women and recruitment was highest in the Central Denmark Region (Table I). Healthcare and employment professionals constituted the largest participant groups, whereas workplace representation was limited. Of the 81 professionals and researchers who participated in brainstorming, 28 were physiotherapists, 20 social workers, 17 physicians, 5 occupational therapists, 3 psychologists, 2 chiropractors, and 6 represented other professions. Employment status varied among the 16 participants with lived experience of LBP (Table I).

**Table I:** Participant characteristics across the group concept mapping phases

Characteristic	Idea generation	Structuring statements		Interpretation and validation
	Brainstorming (*n* = 101)	Sorting (*n* = 23)	Rating (*n* = 36)	Validation workshop (*n* = 11)
Age (years), median (IQR)	45 (39–52)	45 (40–59)	45 (37–60)	38 (33–42)
Women, *n* (%)	78 (77.2)	16 (69.6)	25 (69.4)	9 (81.8)
Stakeholder participants, *n* (%)				
People with lived experience of LBP	16 (15.8)	3 (13.0)	11 (30.6)	3 (27.2)
Researchers	12 (11.9)	4 (17.4)	3 (8.3)	2 (18.2)
Workplace representatives	4 (4.0)	1 (4.4)	1 (2.8)	0
Municipal health professional	12 (11.9)	3 (13.0)	4 (11.1)	2 (18.2)
Municipal employment professional	31 (30.7)	3 (13.0)	5 (13.9)	2 (18.2)
Regional health professional	19 (18.8)	7 (30.4)	9 (25.0)	1 (9.1)
General practitioner	4 (4.0)	1 (4.4)	2 (5.6)	0
Private health professional	3 (3.0)	1 (4.4)	1 (2.8)	1 (9.1)
Geographical location, *n* (%)				
Capital Region of Denmark	9 (8.9)	3 (13.0)	3 (8.3)	1 (9.1)
Central Denmark Region	50 (49.5)	10 (43.5)	17 (47.2)	6 (54.5)
Region Zealand	12 (11.9)	1 (4.4)	4 (11.1)	1 (9.1)
North Denmark Region	14 (13.9)	3 (13.0)	5 (13.9)	1 (9.1)
Region of Southern Denmark	16 (15.8)	6 (26.1)	7 (19.4)	2 (18.2)
Employment status of people with lived experience of LBP, *n* (%)				
Full-time employment	6 (37.5)	0	1 (9.1)	1 (33.3)
Part-time employment	4 (25.0)	1 (33.3)	3 (27.3)	1 (33.3)
On sick leave	3 (18.8)	0	1 (9.1)	1 (33.3)
Disability pension	2 (12.5)	1 (33.3)	3 (27.3)	0
Flex job (subsidized employment)	1 (6.3)	1 (33.3)	3 (27.3)	0

Percentages for employment status are calculated among participants with lived experience of LBP in each phase (brainstorming, *n* = 16; sorting, *n*= 3; rating, *n* = 11; validation workshop, *n* = 3).

IQR: interquartile range; LBP: low back pain.

### Group concept mapping data

As summarized in Fig. 1, brainstorming generated 190 ideas, which were reduced to 125 statements for sorting and rating.

Participants sorted a median of 125 statements (range 96–125) into a median of 8 piles (range 3–14). Stakeholder participants generally assigned high importance ratings, with 81% of all ratings falling within the upper 2 categories (3 or 4 on a 4-point scale).

The concept mapping analysis yielded a stress value of 0.25, indicating an acceptable fit between the similarity matrix and the 2-dimensional point map. The point map with bridging values is presented in Fig. S1. Candidate cluster solutions ranging from 4 to 10 clusters were examined iteratively. A 7-cluster solution was selected for presentation and discussion at the validation workshop because solutions with fewer clusters combined conceptually distinct content, whereas solutions with more clusters appeared to split closely related statements without adding meaningful interpretive value. The clusters in this solution were identified numerically as Clusters 1–7. Median importance ratings varied little across the 7 clusters (3–3.5), indicating that all domains were considered important (Fig. 2).

**Fig. 2:**
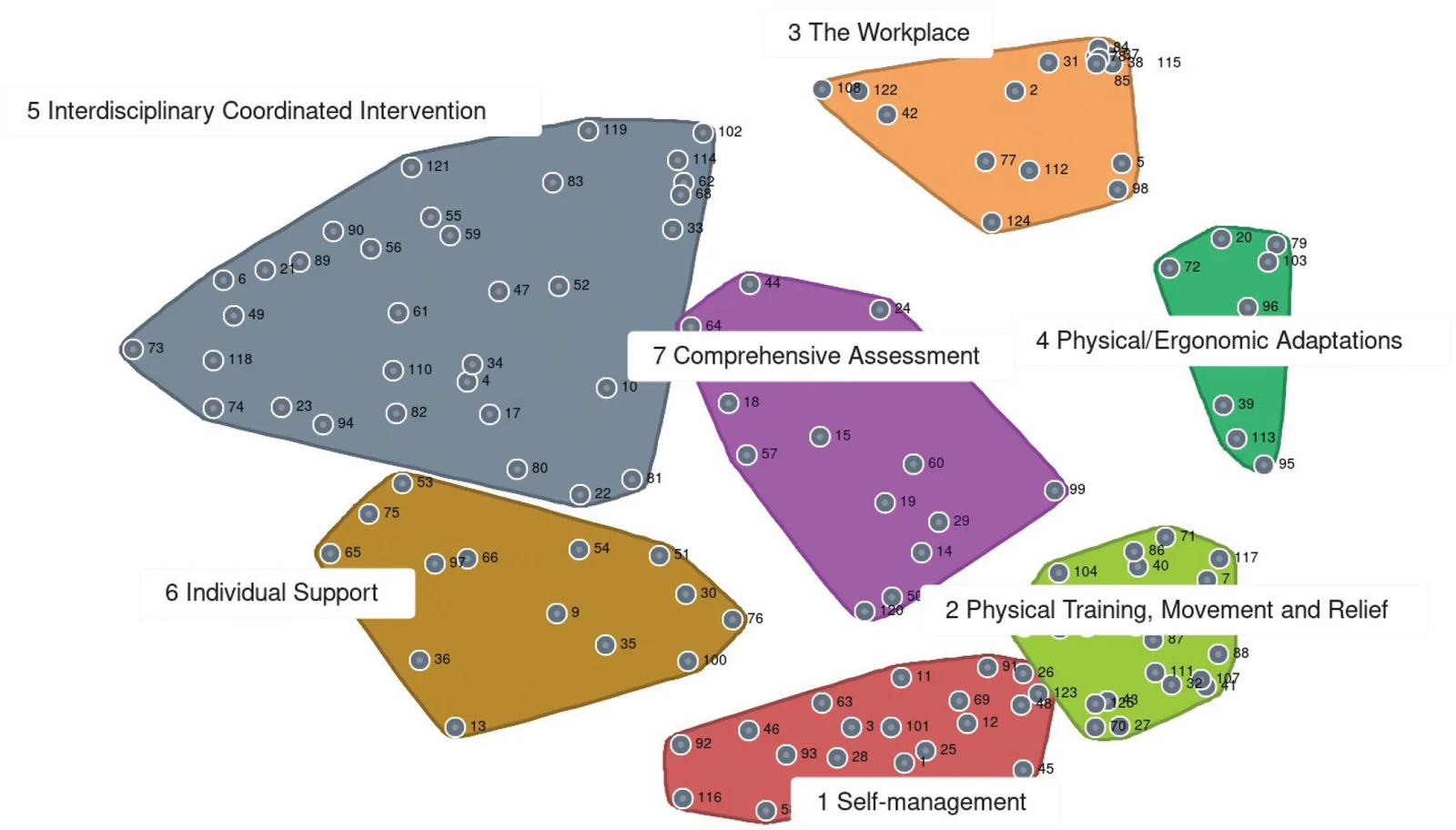
Seven-cluster solution selected for validation. The cluster map shows the spatial relationships between the 125 statements and the 7 clusters presented at the validation workshop. Clusters are identified numerically as Clusters 1–7.

Across the 125 statements, 39 statements (31.2%) had a median importance rating of 4 and primarily concerned self-management, pain understanding and management, everyday activities, coordination, and person-centred rehabilitation. Overall rating patterns were similar between participants with lived experience and professionals. However, those with lived experience placed relatively greater emphasis on support, acknowledgement, and coordination, while professionals emphasized activity-related strategies. Table SII presents all statements, cluster placements, and importance ratings for the total sample and the median ratings of professionals and people with lived experience of LBP.

### Interpretation and validation of the concept map

At the validation workshop, participants reached consensus on retaining the placement of 108 statements (86.4%), while 17 statements (13.6%) were reallocated. The main structural change was dissolving Cluster 4, *Physical/Ergonomic Adaptations*, and redistributing its statements primarily to Cluster 2, *Physical Training, Movement and Relief*, and Cluster 3, The *Workplace*. Three cluster labels were also refined: Cluster 1, *Self-management*, was renamed *Support for Self-management;* Cluster 2, *Physical Training, Movement and Relief*, was renamed *Physical Training and Everyday Movement*; and Cluster 7, *Comprehensive Assessment*, was renamed *Extended Assessment and Support for Treatment*.

Together, these modifications resulted in a final 6-cluster solution. To distinguish the final validated solution from the preliminary 7-cluster solution, its 6 clusters are identified hereafter as Clusters A–F. Table SIII provides further details on the correspondence between the 2 solutions, cluster refinement and statement relocation.

### Final concept map

Table II presents the final validated 6-cluster solution, identified by cluster IDs A–F, with corresponding content descriptions. The clusters represented components of rehabilitation considered important for supporting functioning at home and work among individuals on sick leave due to LBP.

**Table II:** Final validated 6-cluster solution with content descriptions

Cluster ID	Participant-validated cluster label	No. of statements	Content description
A	Support for Self-management	21	Emphasizes strengthening individuals’ capacity for self-management through the development of knowledge, skills, confidence, and a greater sense of control. This includes understanding pain mechanisms, addressing fear-avoidance and emotional responses, adapting to changes in identity and life situation, and maintaining everyday activities. Strategies include pacing, energy management, sleep support, self-regulation, motivation, and opportunities for professional and peer support
B	Physical Training and Everyday Movement	25	Addresses physical activity, exercise, and confidence in movement through consistent training, graded progression, and integration of movement into everyday life. The cluster includes balancing activity and rest, developing body awareness, and reinforcing understanding that movement is safe and beneficial. Professional guidance, reassurance, and therapeutic support may facilitate engagement in continued physical activity
C	The Workplace	21	Positions the workplace as an active context for rehabilitation, including early workplace involvement and graded return to work. It also includes adaptation of work hours, tasks, physical working conditions, workplace demands, and relevant guidance and assistive measures. The cluster also encompasses supportive workplace cultures, involvement of managers and colleagues, and organizational, financial, and policy-related conditions that support sustainable work participation
D	Interdisciplinary Coordinated Intervention	37	Focuses on individualized planning, coordination and alignment across the person, healthcare, employment services, workplaces, relatives, and other relevant stakeholders. This includes shared goals, attention to psychological, social, financial, and broader life circumstances, and recognition of shared responsibility for rehabilitation. The cluster also encompasses support for navigating complex systems, access to relevant services and financial resources, and timely, coherent interventions centred on the individual
E	Individual Support	9	Highlights person-centred and relational support, including being heard, understood, and supported as an individual with unique needs and circumstances. The cluster emphasizes individual goals, meaning-making, motivation, a focus on possibilities and attention to thoughts, worries, psychological strain, and difficult life experiences. It also includes involving relatives and supporting understanding and dialogue within family life and employment services
F	Extended Assessment and Support for Treatment	12	Addresses extended assessment and targeted support when progress is limited or the person’s situation is complex. This includes assessment of functioning at home and at work, rehabilitation and treatment needs, psychological and contextual barriers, and relevant health and social circumstances. The cluster also encompasses reassessment of goals, referral to specialized services when needed, access to treatment and assistive devices, and intensified or adapted support when required

Psychological, social, and everyday-life aspects were distributed across the concept map rather than forming separate clusters. Psychological content included fear avoidance, confidence, motivation, identity, and emotional responses, particularly in Clusters A, E, and F. Social content included support from relatives, colleagues, managers, and services, as well as workplace culture and financial circumstances, particularly across Clusters C–E. Everyday functioning was represented through daily activities, sleep, energy management, family life, and balancing home and work. It was most prominent in Cluster A, Support for Self-management, but was also present across several other clusters.

### Conceptual model of rehabilitation for individuals on sick leave due to low back pain

During the utilization phase, the authors identified Wade’s rehabilitation cycle as a relevant framework for relating the validated clusters to a coordinated and iterative process comprising assessment, goal setting, tailored intervention, and evaluation and adaptation (30). Fig. 3 presents the resulting author-developed conceptual model. Unlike the empirical concept map, the model reorganizes the clusters according to the authors’ interpretation of their functions within the rehabilitation process and should therefore be understood as an analytical synthesis. Fig. 3 shows functioning in everyday life at home and at work is revealed as an overarching focus of rehabilitation, reflecting the study’s focus prompt, rather than as an outcome demonstrated by the present study.

**Fig. 3:**
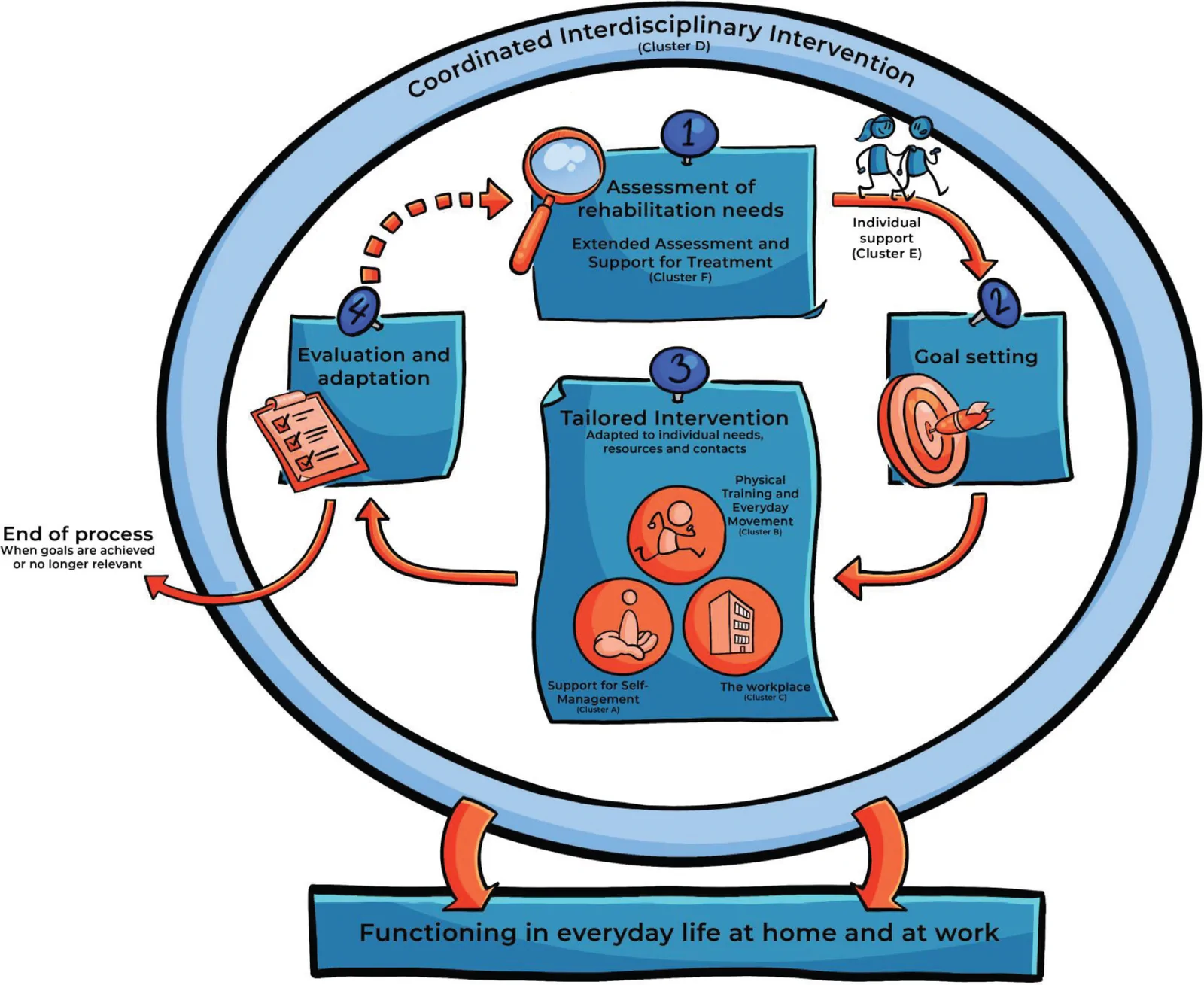
Author-developed conceptual model of rehabilitation for individuals on sick leave due to low back pain. The organization into assessment, goal setting, intervention, and evaluation and adaptation was informed by Wade’s rehabilitation cycle (30). Functioning in everyday life at home and at work reflects the scope of the focus prompt. Cluster placement represents the authors’ theoretical interpretation of the empirical concept-map findings. *Interdisciplinary Coordinated Intervention* (Cluster D) indicates coordination between relevant professionals, services, and sectors when required. The professionals involved and the emphasis and intensity of the intervention domains may vary according to individual needs, resources, and context.

Cluster D, *Interdisciplinary Coordinated Intervention,* was positioned as an overarching and integrating element. This reflects the cluster’s emphasis on alignment between the individual and relevant professionals, services, and sectors when coordination is required. Cluster E, *Individual Support,* was conceptualized as a supportive and relational element throughout the process, represented in the model by the individual and professional moving together through the rehabilitation process. Cluster F, *Extended Assessment and Support for Treatment*, was positioned in relation to the assessment phase, reflecting the importance of comprehensive assessment and support when situations are complex, or progress is limited, rather than a requirement for all individuals. This may include assessment of functioning, rehabilitation needs, treatment needs, and contextual factors as a foundation for goal setting and tailoring relevant rehabilitation interventions. The tailored intervention phase integrates the 3 overlapping domains: Cluster A, *Support for Self-management*; Cluster B, *Physical Training and Everyday Movement*; and Cluster C, *The Workplace*. Their overlap illustrates that these domains may interact, while their emphasis and intensity may be adapted to individual needs, resources, and context over time.

## DISCUSSION

This study identified and prioritized stakeholder-defined components of rehabilitation considered important for supporting functioning among individuals on sick leave due to LBP. Collectively, the findings suggest rehabilitation for people on sick leave due to LBP as a coordinated, multicomponent process extending across healthcare, employment services, workplaces, and everyday life contexts.

The utilization phase extended the empirical concept map by relating the validated clusters to an established rehabilitation framework. Together, the concept map and conceptual model provide a stakeholder- and theory-informed foundation to be integrated with research evidence and contextual inquiry during subsequent programme theory and intervention development.

Although the focus prompt did not explicitly refer to biopsychosocial rehabilitation, the stakeholder-generated statements encompassed physical, psychological, social, and work-related aspects of functioning and rehabilitation. The findings can therefore be interpreted as consistent with current recommendations for multidisciplinary biopsychosocial rehabilitation for people with LBP (12–14, 31). Systematic reviews of multidisciplinary biopsychosocial rehabilitation suggest small improvements in pain and disability, whereas effects on return to work and sickness absence remain uncertain (12, 14, 15). The certainty of this evidence varies, and concerns have been raised as to the trustworthiness of some contributing trials (32). Our previous systematic review used the TIDieR checklist (33) to systematically describe the content and delivery of 3 identified workplace-integrated biopsychosocial rehabilitation programmes (16). The review revealed a limited evidence base and a relatively narrow operationalization of biopsychosocial rehabilitation (16). The evaluated programmes primarily combined workplace interventions with graded activity or exercise. Psychological principles were largely integrated into physical activity components, whereas broader psychological, social, and everyday-life dimensions received limited attention (16). The extent and nature of interdisciplinary collaboration also varied considerably across programmes (16). Similar challenges in intervention specification and component integration have been reported in broader reviews (12, 14).

The present study addresses these conceptual and content-related gaps by identifying self-management, person-centred and relational support, extended assessment, and coordination across services and sectors alongside physical and workplace-related components. However, stakeholder prioritization reflects clinical experience and contextual knowledge rather than evidence of effectiveness or causal mechanisms of the identified components. Components that were not prominent in the concept map, including nutrition and systematic comorbidity management, should not be interpreted as unimportant. The relevance of both identified and less prominent components of the cluster map should be assessed alongside research evidence and clinical guidance during subsequent programme theory and intervention development. The findings nevertheless support the interpretation of rehabilitation for people on sick leave due to LBP as a complex intervention in which outcomes emerge through interactions between multiple components, stakeholders, and contextual factors (24, 31).

This complexity is particularly reflected in Cluster D, *Interdisciplinary Coordinated Intervention*. Stakeholders emphasized alignment and collaboration between healthcare professionals, employment services, and workplaces. This interdisciplinary coordination appeared to function not merely as an organizational prerequisite but as an integrative process linking otherwise fragmented components across sectors and contexts. This aligns with the complex intervention model proposed by Nieminen et al., in which coordination across professionals and sectors influences how biopsychosocial components are delivered, combined, and adapted over time (34). From a broader rehabilitation perspective, coordination may therefore be understood as an explicit function involving alignment of goals, communication, monitoring, continuity, and adaptation across components and settings (30, 35). The findings do not determine whether this function should be undertaken by 1 professional or shared across a rehabilitation team. Its organization requires contextual specification and evaluation during subsequent intervention development.

Cluster E, *Individual Support,* and Cluster F, *Extended Assessment and Support for Treatment*, similarly appeared to operate as adaptive processes rather than discrete components. Fig. 3 depicts rehabilitation as an iterative, person-centred process involving assessment of rehabilitation needs, goal setting, tailored intervention, and ongoing evaluation and adaptation (30, 35). Stakeholders emphasized the importance of understanding the person within the context of everyday life to tailor rehabilitation services to changing needs and phases. In this way, assessment and individual support appeared as processes operating throughout rehabilitation rather than only as preparatory steps. Whereas Clusters D–F primarily concern how rehabilitation may be coordinated, adapted, and sustained over time, Clusters A–C focused more on domain-specific rehabilitation components, particularly physical activity and exercise, self-management, and workplace participation. However, although explicitly embedded in the focus prompt, functioning in everyday life at home did not emerge as a distinct aspect of stakeholders’ conceptualization of rehabilitation for individuals on sick leave due to LBP. Instead, aspects of everyday life appeared to be distributed across multiple clusters, including Cluster A, *Support for Self-management*; Cluster E, *Individual Support*; and Cluster F, *Extended Assessment and Support for Treatment*.

One possible explanation is that physical activity and exercise, self-management, and workplace-related approaches are among the most consistently emphasized components across existing rehabilitation approaches for people with LBP (13, 16, 18). These more established rehabilitation components may have shaped how stakeholders interpreted, sorted, and prioritized the statements. By contrast, everyday functioning outside work may be less clearly established as a distinct rehabilitation domain than work participation. Whereas work participation is often structured around identifiable actors, procedures, and intervention targets, previous qualitative research suggests that the everyday consequences of LBP are dispersed across routines, relationships, social identity, and ongoing adaptations in daily life (10, 19, 36, 37). Difficulties related to domestic routines, family roles, rest, energy management, identity, and ordinary daily activities may therefore have been experienced as important, but less readily recognized as a distinct rehabilitation component. A further explanation may be that the twofold focus on everyday life outside work and participation at work led stakeholders to prioritize more institutionally visible and actionable rehabilitation domains, particularly those connected to return-to-work processes.

Together, these findings suggest that future biopsychosocial rehabilitation approaches for people on sick leave due to LBP should more explicitly conceptualize and operationalize support for everyday functioning alongside more clearly consolidated components related to physical activity and exercise, self-management, and workplace participation. Future studies should therefore examine everyday life as a primary focus, including how people on sick leave due to LBP manage daily activities, family roles, routines, identity changes, and the relationship between functioning outside work and participation at work.

### Strengths and limitations

The findings were generated within the Danish welfare system and may be transferable to settings with similar healthcare, social-protection, and employment structures. The broader principles of person-centred assessment, adapted support, workplace involvement, and coordination may have wider relevance, but their operationalization depends on local resources, employment conditions, social protection, and workplace capacity. Generalizability may therefore be more limited in low- and middle-income settings and requires cross-cultural and contextual adaptation rather than direct replication.

A key strength of the present study was that the focus prompt explicitly encompassed functioning at home and work, reflecting a holistic understanding of rehabilitation and functioning (8). Another strength was the inclusion of multiple stakeholder groups across professional and organizational settings, national recruitment across all 5 Danish regions, and participation of 101 stakeholders. This strengthened the study by incorporating rehabilitation perspectives from various arenas covered by the rehabilitation process.

Several limitations should be considered. First, the twofold focus on functioning at home and work participation may have reduced response specificity. This may partly explain why aspects related to functioning in everyday life at home appeared less cohesive within the concept map. Second, stakeholder representation was uneven. The predominance of healthcare and employment professionals may have favoured institutionally recognized components over domestic activities, family roles, and other aspects of everyday life outside work. However, this study cannot determine the extent of this influence.

Workplace representation was particularly limited. We did not collect data on reasons for non-participation. However, indirect recruitment, competing operational demands, and the time required for the multi-stage GCM process may have constrained participation. Consequently, workplace perspectives may be insufficiently represented despite the prominence of workplace-related content. Participants’ familiarity with specific rehabilitation approaches was not systematically assessed and may have influenced the perspectives represented. The relatively high attrition during the structuring phases should also be acknowledged as a limitation. Attrition during sorting and rating is a recognized challenge in GCM studies, particularly because the sorting task is cognitively demanding and time-consuming (27, 28). Nevertheless, key stakeholder perspectives remained represented, and the number completing the structuring tasks was within the recommended range of 20–40 participants for producing interpretable and stable GCM cluster solutions (27, 28). Uneven stakeholder representation should be considered when interpreting the map. Finally, the author-developed conceptual model cannot guide implementation without further development but is intended to inform subsequent programme theory and intervention development for people on sick leave due to LBP.

### Conclusion

The stakeholder priorities encompassed physical, psychological, social, and work-related aspects of rehabilitation, consistent with a biopsychosocial perspective. The validated clusters and author-developed conceptual model recommend rehabilitation as a coordinated, interdisciplinary, adaptive, and person-centred process integrating components across phases, sectors, and everyday-life contexts. Although functioning at home was represented across several clusters, it did not emerge as a distinct rehabilitation domain, indicating a need to further specify how functioning at home can be assessed, addressed, and integrated alongside work participation. Together, the concept map and conceptual model provide a stakeholder- and theory-informed foundation that can be integrated with existing research evidence in subsequent programme theory and intervention development.

## Supplementary Material






