# Supplementary material for: STAKEHOLDER PRIORITIES FOR REHABILITATION TO SUPPORT FUNCTIONING AT HOME AND WORK AMONG PEOPLE ON SICK LEAVE DUE TO LOW BACK PAIN: A GROUP CONCEPT MAPPING STUDY
Source: J Rehabil Med. 2026 Nov 13;58:46346. doi: 10.2340/jrm.v58.46346 (PMC13760672; doi:10.2340/jrm.v58.46346)
Supplement: Supplementary file 1 [file JRM-58-46346-s1.pdf]

Checklist items for reporting Trochim's concept mapping research (ConMapT)

Manuscript: Stakeholder priorities for rehabilitation to support functioning at home and work among people on sick leave due to low back pain: a group concept mapping study

Prepared for submission to Journal of Rehabilitation Medicine

Instructions: Complete the final column with final page numbers or section references after the manuscript has been paginated. Where an item is not applicable, state "Not applicable" and briefly justify if needed.

Heading	Section and topic	Item	Checklist item	Location in manuscript / supplementary material
Title	Title	**1**	State that the manuscript is reporting a concept mapping study.	*Title page*
Abstract	Abstract	**2a**	Describe the core problem (focus) of the research.	*Abstract, objective*
		**2b**	State concept mapping as the study methodology and list the phases of concept mapping undertaken.	*Abstract, design and methods*
		**2c**	Indicate where the fieldwork was conducted.	*Abstract, design*
		**2d**	State the stakeholder groups involved in the research. Report the total number of participants involved in the study.	*Abstract, subject/patients*
		**2e**	Give a brief description of the final concept map that should include cluster labels.	*Abstract; Results*
Background	Rationale	**3**	Provide a sound scientific rationale for the study.	*Introduction*
	Aim	**4**	State the study aim(s).	*Introduction*
	Methodological justification	**5a**	Provide a justification for using concept mapping to address study aim(s).	*Introduction; Methods*
	Methodological justification	**5b**	State which of the six phases of concept mapping were undertaken. Justify why phase(s) of concept mapping were omitted.	*Methods*
Phase 1: Preparation	Determining the focus prompt	**6a**	State the focus prompt for the study.	*Methods*
		**6b**	Describe the process for developing the focus prompt and, if applicable, how stakeholders were involved.	*Methods*
	Participants	**7a**	Justify why each stakeholder group was selected.	*Methods*
		**7b**	State the eligibility criteria for each stakeholder group.	*Methods*
		**7c**	Describe how study participants were recruited.	*Methods*
		**7d**	Provide a rationale for the number of participants at each phase of concept mapping.	*Methods*
		**8a**	Describe the flow of participants (ideally as a flow diagram) through each phase of concept mapping.	*Results, Figure 1*
		**8b**	Tabulate and describe the demographic and clinical (if relevant) characteristics of each of the stakeholder groups.	*Results, Table I*
	Public involvement	**9**	Explain how patients and/or the public were involved in each phase of the research, from conception through dissemination.	*Methods. Stakeholder advisory panels contributed to the study aim, focus prompt, and recruitment strategy.*
	Software package	**10**	State which software package was used in each phase of concept mapping.	*Methods*
Phase 2: Generating the ideas	Brainstorming	**11a**	State all methods used for idea generation, for example brainstorming or literature review.	*Methods*
		**11b**	Describe in detail how brainstorming sessions were conducted and recorded, for example individual interviews or focus groups.	*Methods*
		**11c**	Explain how data from the brainstorming sessions was turned into statements.	*Methods*
		**11d**	Describe factors that may have influenced idea generation, for example group dynamics.	*Methods; Limitations*
		**11e**	Report the number of statements generated from each approach to idea generation, for example literature review or brainstorming.	*Results*
	Statement reduction	**12a**	Describe and justify the procedure for statement reduction, for example eliminating overlapping statements or combining similar statements.	*Methods*
		**12b**	Describe to what extent participant wording was retained in the statements.	*Methods*
		**12c**	Describe how stakeholders were involved in statement reduction.	*Not applicable*

Heading	Section and topic	Item	Checklist item	Location in manuscript / supplementary material
		**13**	Report the final number of statements following statement reduction. Ensure a complete list of the statements is available, for example as a supplementary document or data file.	*Results; Supplementary Table SII*
Phase 3: Structuring the statements	Clustering and rating	**14a**	Describe the procedures followed for structuring statements, including clustering and rating tasks.	*Method,*
		**14b**	Provide information about the instructions to participants as to how to complete the clustering and rating tasks.	*Methods*
		**15**	Report the average (mean, median) number of clusters generated by the participants.	*Results*
		**16**	Report the mean rating score for each statement. Consider reporting as a supplementary document or data file.	*Results; Supplementary Table SII (median ratings are reported because the rating scale was ordinal)*
Phase 4: Concept mapping analysis	Data cleaning	**17a**	Describe procedures for data checking and cleaning.	*Methods*
		**17b**	Describe procedures for handling missing data.	*Methods*
	Statistical procedures	**18a**	Specify what statistical procedures were undertaken to generate candidate concept maps.	*Methods*
		**18b**	Specify the statistical tests undertaken to determine the validity of the concept map, for example stress value or split-half reliability test.	*Methods; Results*
		**18c**	Describe any additional analyses not in the study protocol.	*Not applicable*
Phase 5: Interpreting the map	Data interpretation process	**19a**	State how the final concept map was selected.	*Methods; Results*
		**19b**	Provide a description of how cluster labels on the final concept map were determined.	*Methods; Results*
		**19c**	Describe how stakeholders provided feedback on the final concept map.	*Methods; Results*
	Description of the final concept map	**20a**	Describe the final concept map.	*Results; Table II*
		**20b**	State and justify any post hoc adjustments to the concept map, for example moving statements between or combining clusters.	*Results; Supplementary Table SIII*
		**20c**	Describe each cluster, including illustrative statements, in the final concept map.	*Results; Table II*
	Rating data	**21**	If applicable, describe the go-zone, including examples of statements in each quadrant, or pattern match (ladder graph).	*Not applicable*
Phase 6: Utilization	Utilization	**22**	Provide a statement on how the concept map will be utilised.	*Discussion; Conclusion*
Discussion	Discussion	**23**	Locate the concept map within the context of existing evidence.	*Discussion*
Limitations	Limitations	**24**	State all study limitations, including those that may have arisen because of deviations from the study protocol.	*Discussion*
Ethics	Ethics	**25a**	State which ethics committee or Institutional Review Board reviewed the study, including the ethics committee reference number.	*Ethics declarations*
		**25b**	Describe the ethical issues in the study.	*Ethics declarations; Methods*
		**25c**	Describe the procedure for obtaining informed consent from the study participants.	*Methods; Ethics declarations*
		**25d**	State how participants were compensated for taking part in the study.	*Methods*
Conclusion	Conclusion	**26**	Summarise the key findings from the study.	*Conclusion*
Registration and protocol	Registration and protocol	**27a**	Give details of the registration status of the study, including registry and registration number.	*Ethics declarations and trial registry information*
		**27b**	State how the study protocol can be accessed.	*No study protocol was published*
		**27c**	List any deviations from the study protocol.	*No protocol deviations were applicable because no published protocol existe*

Source: ConMapT reporting guideline checklist for Trochim's concept mapping research, developed by Pantha, Jones and Gray (2025).
