# Supplementary material for: STAKEHOLDER PRIORITIES FOR REHABILITATION TO SUPPORT FUNCTIONING AT HOME AND WORK AMONG PEOPLE ON SICK LEAVE DUE TO LOW BACK PAIN: A GROUP CONCEPT MAPPING STUDY
Source: J Rehabil Med. 2026 Nov 13;58:46346. doi: 10.2340/jrm.v58.46346 (PMC13760672; doi:10.2340/jrm.v58.46346)
Supplement: Supplementary file 2 [file JRM-58-46346-s2.pdf]

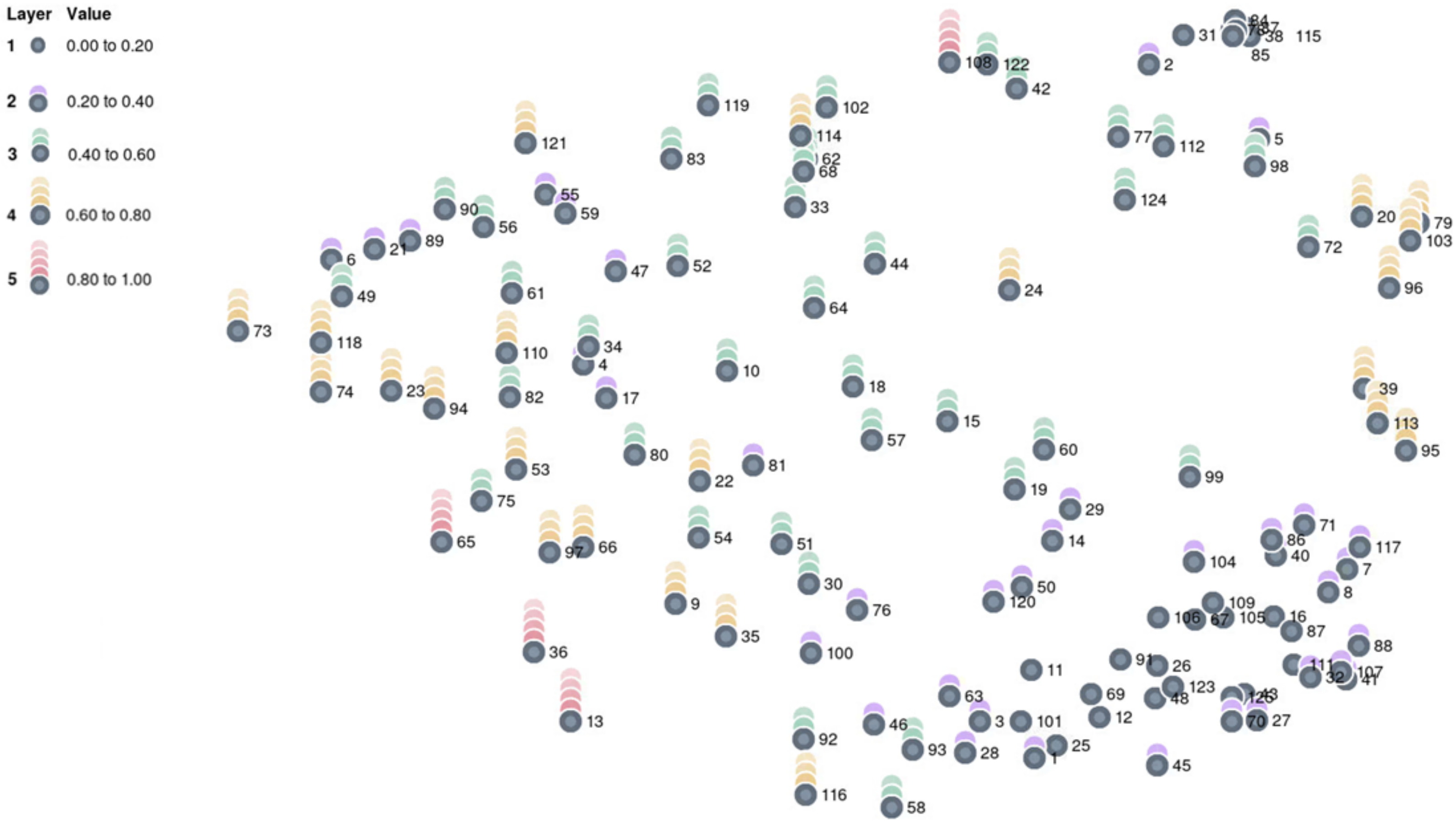

Figure S1. Point map with bridging values from the candidate seven-cluster solution
