# Supplementary material for: STAKEHOLDER PRIORITIES FOR REHABILITATION TO SUPPORT FUNCTIONING AT HOME AND WORK AMONG PEOPLE ON SICK LEAVE DUE TO LOW BACK PAIN: A GROUP CONCEPT MAPPING STUDY
Source: J Rehabil Med. 2026 Nov 13;58:46346. doi: 10.2340/jrm.v58.46346 (PMC13760672; doi:10.2340/jrm.v58.46346)
Supplement: Supplementary file 3 [file JRM-58-46346-s3.pdf]

Supplementary overview

This supplementary material provides additional details on the group concept mapping (GCM) process and results. Supplementary Table SI shows examples of the transformation of original brainstorming responses into final statements.

Supplementary Table SII presents the reduced set of 125 statements included in the GCM sorting and rating phases, organised according to the candidate seven-cluster solution prior to stakeholder interpretation and validation. The statements were generated in response to the focus prompt:

"For a person on sick leave due to low back pain to have the best possible everyday life, both at home and at work, in my experience, there is a need for ..."

All statements were originally generated in Danish and subsequently translated into English by the authors for reporting purposes. The original Danish version of the statements, as well as the full unreduced list of statements generated during the brainstorming phase, can be obtained from the corresponding author upon reasonable request. The total rating sample included all participants who completed the rating task. Subgroup ratings are shown for people with lived experience of LBP and professionals; participants not included in these two subgroup categories are included only in the total rating.

Supplementary Figure S1 presents the point map with bridging values from the candidate seven-cluster solution. Supplementary Table SIII summarises adjustments made to the concept map during the validation workshop, including cluster renaming, restructuring, and statement relocation.

Table SI. Examples of the transformation of original brainstorming responses into final statements

Original brainstorming response[1]	Final statement(s)	Processing undertaken	Final cluster
"Graded return to work, not only in terms of hours but also work tasks."	**No. 2:** "Graded return to work, both in terms of hours and work tasks."	Minor linguistic revision	Cluster C: *The Workplace*
"There is a need for the workplace to be actively involved early in the process, so that realistic solutions can be found that make it possible to maintain contact with work."	**No. 5:** "Early active involvement of the workplace so that realistic solutions can be found that make it possible to maintain contact with work."	Removal of wording repeating the focus-prompt stem; original meaning retained	Cluster C: *The Workplace*
"The individual should be supported in seeing possibilities rather than limitations. It is also important to involve significant others or relatives, as this may create greater understanding of the individual's challenges."	**No. 35:** "Supporting the individual to see possibilities rather than limitations." **No. 36:** "Recognising the importance of involving significant others or relatives, as this may create greater understanding of the individual's challenges."	Response containing two distinct ideas split into two statements	Cluster E: *Individual Support*
"Collaboration between the job centre/social worker/physiotherapist/doctor and other relevant professionals."[2]	**No. 21:** "Collaboration between the job centre/social worker/physiotherapist/doctor and other relevant professionals."	Substantially overlapping responses merged, and one duplicate removed; wording retained	Cluster D: *Interdisciplinary Coordinated Intervention*

[1] Original responses were provided in Danish and translated into English for presentation

[2] Two substantially overlapping responses expressed this idea.

Table SII. Reduced set of statements included in the group concept mapping sorting and rating phases (n = 125)

Cluster 1: Self-management

		Median (range)			
**No.**	**Statement**	**Total (n = 36)**	**People with lived experience (n = 11)**	**Professionals (n = 21)**	**Median difference**
1	energy management and activity balance.	3 (1–4)	4 (2–4)	3 (1–4)	1
3	pedagogical phrases/metaphors. For example, before starting a work task or daily activity, the person should be able to answer “yes” to the following: “I am not harming myself, and I will not pay the price later.”	3 (1–4)	3 (1–4)	2 (1–4)	1
11	attention to the psychological impact of experiencing loss, for example loss of freedom, future dreams, self-image, and identity. Support may be needed to reorient and formulate new goals and values.	3 (2–4)	4 (3–4)	3 (2–4)	1
12	support for the person with pain to experience a greater sense of control over their pain, for example through methods to relieve pain or distract themselves from it.	3 (2–4)	4 (2–4)	3 (2–4)	1
25	teaching principles rather than only methods; otherwise, people cannot truly master anything. “Master the principle and you will never need another method.”	3 (1–4)	3.5 (1–4)	3 (1–4)	0.5
26	helping people understand that discomfort and pain are not the same, and that any exercise that improves back function may also cause temporary discomfort.	3 (1–4)	3 (1–4)	3 (1–4)	0
28	support to improve sleep.	3 (1–4)	4 (3–4)	3 (1–4)	1
45	motivation on the part of the individual to be physically active through rehabilitation exercises and walking.	4 (2–4)	4 (2–4)	4 (2–4)	0
46	support for self-management. A person with low back pain should be given relevant knowledge and tools to manage everyday life with pain, with as little fear of movement and the future as possible.	4 (2–4)	4 (3–4)	4 (2–4)	0
48	patient education focusing on energy management, pain management, and knowledge. The aim is to provide tools to take responsibility for one’s own life.	4 (2–4)	4 (3–4)	3 (2–4)	1
58	use of a weekly schedule to assess sustainability across family life, treatment, exercises, work, etc.	2 (1–4)	2 (1–4)	2 (1–4)	0
63	group sessions led by a psychologist on the importance of biopsychosocial factors in pain.	3 (1–4)	3 (1–4)	3 (1–4)	0

69	coaching or motivational groups to help people get through the pain and/or learn to manage it.	3 (1–4)	3 (2–4)	3 (1–4)	0
91	conversations about the psychological impact of having experienced such severe pain. It changes a person. One may develop anxiety/fear of experiencing it again and become hypervigilant to bodily signals.	3 (2–4)	4 (2–4)	3 (2–4)	1
92	contact with peers in a similar situation with whom one can identify. Conversations with peers after rehabilitation are important.	3 (1–4)	3 (1–4)	2 (1–4)	1
93	listening to one's body and setting limits if rest is needed because of back pain. Taking the time needed to recover.	3 (1–4)	4 (3–4)	3 (1–4)	1
101	motivational support for pain management.	4 (2–4)	4 (2–4)	3 (2–4)	1
116	maintaining daily activities.	4 (1–4)	4 (3–4)	4 (1–4)	0
123	addressing fear avoidance and awareness of it not only during exercise but also in everyday activities: "It hurt when I lifted a liter of milk, so now I avoid doing it."	4 (1–4)	4 (2–4)	3 (1–4)	1

Cluster 2: Physical Training, Movement and Relief

		**Median (range)**			
**No.**	**Statement**	**Total (n = 36)**	**People with lived experience (n = 11)**	**Professionals (n = 21)**	**Median difference**
7	focus on acute pain management and, as far as possible, non-pharmacological maintenance treatment for non-inflammatory chronic low back pain.	3 (1–4)	3 (2–4)	3 (1–4)	0
8	assessment of how low back pain reduces functioning, and alignment of expectations regarding whether and how functioning can be improved to an acceptable and individualised level.	3 (2–4)	4 (3–4)	3 (2–4)	1
16	helping the person understand their pain as biopsychosocial rather than only biomechanical.	4 (1–4)	4 (1–4)	4 (2–4)	0
27	consistent and stable exercise. Carrying out exercise regularly and consistently produces results. The specific choice of exercises is often less important than gradual progression and adaptation.	3 (2–4)	3 (2–4)	3 (2–4)	0
32	an appropriate balance between training/demands and recovery/rest.	4 (3–4)	4 (3–4)	4 (3–4)	0

40	support and guidance to help the person "move on/get started again." They need professional help to navigate what they are allowed to do and, fundamentally, to learn that they can do most things.	3 (2–4)	4 (2–4)	3 (2–4)	1
41	maintenance exercise.	3 (1–4)	4 (2–4)	3 (1–4)	1
43	better understanding that low back pain does not improve by lying/sitting all day, but also not by being active all the time.	4 (2–4)	4 (2–4)	4 (3–4)	0
67	helping people believe that it is possible to live with back pain.	4 (1–4)	4 (2–4)	4 (1–4)	0
70	offering massage alongside rehabilitation exercise.	1 (1–4)	4 (1–4)	1 (1–2)	3
71	providing evening back rehabilitation services, like fitness programs, so that people can improve their health outside working hours.	3 (1–4)	4 (1–4)	3 (1–4)	1
86	sufficient time in the healthcare encounter to listen, assess, and advise.	4 (1–4)	4 (3–4)	4 (1–4)	0
87	providing the person with low back pain with knowledge that: almost everyone experiences low back pain from time to time; in most cases it is bothersome and painful but not dangerous; there is no single "right" training program or exercise- the important thing is to keep moving.	4 (1–4)	4 (1–4)	4 (2–4)	0
88	whole-body exercise delivered with close follow-up<br><br>by therapists who also prepare the individual to maintain their level of functioning independently.	3 (2–4)	4 (2–4)	3 (2–4)	1
104	therapeutic conversations in severe cases involving strong reactions to pain, fear of movement, depressive symptoms, or fear related to everyday life and work.	3 (1–4)	4 (1–4)	3 (2–4)	1
105	thorough conversations about pain: chronic pain does not necessarily indicate tissue damage but may reflect nervous system changes that can be reduced through activity.	4 (2–4)	4 (2–4)	4 (2–4)	0
106	ongoing opportunity to discuss and reflect on pain management with professionals such as physiotherapists, occupational therapists, or support workers.	3 (1–4)	3 (3–4)	3 (1–4)	0
107	help with exercising the back.	3 (1–4)	4 (2–4)	3 (1–4)	1
109	thorough reassurance that the condition is not dangerous and typically improves over time with activity and healthy habits.	4 (2–4)	3 (2–4)	4 (2–4)	-1
111	encouraging the individual to remain as active as possible to avoid deterioration.	4 (1–4)	4 (1–4)	3 (1–4)	1

117	practical and advisory support enabling the person on sick leave to participate in physical exercise.	3 (2–4)	4 (2–4)	3 (2–4)	1
125	focus on body awareness through guided sensory integration, supporting perception of bodily boundaries and regulation of tension.	3 (1–4)	4 (1–4)	2 (1–3)	2

Cluster 3: The Workplace

		**Median (range)**			
**No.**	**Statement**	**Total (n = 36)**	**People with lived experience (n = 11)**	**Professionals (n = 21)**	**Median difference**
2	graded return to work, both in terms of hours and work tasks.	4 (2–4)	4 (2–4)	3 (2–4)	1
5	early active involvement of the workplace so that realistic solutions can be found that make it possible to maintain contact with work.	4 (1–4)	4 (1–4)	4 (3–4)	0
31	guidance for the workplace on how best to support the person on sick leave.	3 (2–4)	4 (3–4)	3 (2–4)	1
37	a workplace culture in which it is legitimate to talk about pain and the need for support without it being perceived as weakness.	3 (1–4)	3 (2–4)	3 (1–4)	0
38	a supportive work environment in which colleagues and managers show understanding and help create a sense of security around returning to work.	4 (1–4)	4 (3–4)	3 (1–4)	1
42	assistive devices/personal assistance through compensatory schemes to help maintain labour market attachment.	3 (2–4)	3 (3–4)	3 (2–4)	0
77	allowing the person with back pain time, without managers or others attempting to accelerate return to work.	3 (1–4)	3 (2–4)	3 (1–4)	0
78	colleagues being able to understand back problems and the pain associated with them.	3 (1–4)	4 (2–4)	3 (1–4)	1
84	a proactive and accommodating workplace with clear guidelines, competencies, and communication aligned with clinical guidelines (and communication from clinicians/municipality).	3 (1–4)	3.5 (2–4)	3 (1–4)	0.5
85	an accommodating workplace—in terms of time, physical setup, and task allocation.	3 (1–4)	4 (3–4)	3 (1–4)	1
98	guidance both in the workplace and at home.	3 (1–4)	4 (2–4)	3 (1–4)	1
108	support from the surrounding environment (family, friends, and especially the workplace).	3 (2–4)	4 (2–4)	3 (2–4)	1

112	thorough assessment of whether work environment exposures are triggering and/or aggravating the condition.	3 (2–4)	4 (2–4)	3 (2–4)	1
115	adapting the workplace to the patient's physical and psychological capacities.	3 (1–4)	4 (3–4)	3 (1–4)	1
122	considering financial incentives for companies that contribute to bringing the person back to work or retaining them in employment.	3 (1–4)	3 (1–4)	2 (1–4)	1
124	knowledge about the meaning and value of work.	3 (1–4)	4 (1–4)	3 (1–4)	1

Cluster 4: Physical/Ergonomic Adaptations

		**Median (range)**			
**No.**	**Statement**	**Total (n = 36)**	**People with lived experience (n = 11)**	**Professionals (n = 21)**	**Median difference**
20	stopping the use of ergonomic measures/lifting techniques in workplaces as the main approach, and instead providing education based on current knowledge about the back.	3 (1–4)	3 (1–4)	3 (1–4)	0
39	gradual progression of load/activity.	4 (2–4)	3 (2–4)	4 (2–4)	-1
72	variation in working positions.	3.5 (2–4)	4 (3–4)	3 (2–4)	1
79	time for exercise, even if it takes place during working hours.	3 (1–4)	3 (1–4)	3 (1–4)	0
95	rest.	3 (1–4)	4 (3–4)	3 (1–4)	1
96	being able to stand, walk, and sit during the day.	3 (2–4)	4 (4–4)	3 (2–4)	1
103	occupational therapy guidance and assessment in relation to workplace design and adjustments.	3 (2–4)	4 (3–4)	2 (2–4)	2
113	exercise tailored to work functions.	3 (1–4)	4 (1–4)	3 (1–4)	1

Cluster 5: Interdisciplinary Coordinated Intervention

		**Median (range)**			

No.	Statement	Total (n = 36)	People with lived experience (n = 11)	Professionals (n = 21)	Median difference
4	early, individualised, holistic, and interdisciplinary rehabilitation.	4 (2–4)	4 (3–4)	4 (2–4)	0
6	individualised and targeted medical certificates requested by the municipality; standardised and generic questions yield standardised and generic answers.	3 (1–4)	4 (1–4)	2 (1–4)	2
10	an individually tailored intervention that may look quite different from one person to another.	4 (1–4)	4 (3–4)	4 (1–4)	0
17	taking a holistic view of the patient when developing the rehabilitation plan.	4 (2–4)	4 (3–4)	4 (2–4)	0
21	collaboration between the job center/social worker/physiotherapist/doctor and other relevant professionals.	4 (2–4)	4 (3–4)	3 (2–4)	1
22	awareness that many individuals strongly wish to succeed with, for example, physical activity, but are unable to do so because they are already overwhelmed in trying to structure and sustain concentration on their training, etc.	3 (1–4)	4 (3–4)	3 (1–4)	1
23	attention to the possibility that the person may lack the financial means to obtain appropriate help, for example from a physiotherapist. Many are denied one-off financial support and stop seeking other options afterwards.	3 (1–4)	4 (1–4)	3 (1–4)	1
33	support from healthcare professionals, family, and employer to help navigate a difficult period.	3 (2–4)	4 (3–4)	3 (2–4)	1
34	financial support for services such as chiropractic care, physiotherapy, osteopathy, etc.	3 (1–4)	3.5 (3–4)	2 (1–4)	1.5
47	developing an individual plan together with the person and relevant stakeholders, focusing on the person's resources for returning to the labour market.	4 (2–4)	4 (2–4)	4 (2–4)	0
49	making it as easy as possible for people to access the help they need; organising the system around the person rather than expecting them to navigate a system built on professional assumptions.	4 (1–4)	4 (3–4)	3 (1–4)	1
52	clear expectation alignment among clinician, relatives, adviser, and employer.	3.5 (2–4)	4 (3–4)	3 (2–4)	1
55	the occupational support should not focus solely on the individual's own responsibility to set limits (many are unable to do so), but advisers should be given tools to understand this as a shared effort. Dialogue tools, question guides, etc., may support this.	3 (1–4)	4 (2–4)	2 (1–4)	2
56	systematic screening in the occupational support, early in the case, for personality traits/coping strategies such as overdoing, perfectionism, and conscientiousness.	2 (1–4)	2 (1–4)	2 (1–4)	0

59	increasing awareness among advisers and job consultants that pain can also give rise to psychological/cognitive work accommodations, not only physical accommodations.	4 (2–4)	4 (2–4)	3 (2–4)	1
61	systematic assessment by advisers, in new cases, of whether psychosocial factors play a role, with access to consultation with a psychologist or medical adviser regarding the intervention plan.	3 (1–4)	3 (1–4)	3 (1–4)	0
62	strong focus among advisers on the person's condition after the end of the working day, to assess whether the solution is sustainable.	3 (1–4)	3 (1–4)	3 (1–4)	0
68	coordinated intervention between the health sector and the workplace, with shared goals.	3.5 (3–4)	4 (3–4)	3 (3–4)	1
73	security regarding finances.	3 (1–4)	4 (2–4)	3 (1–4)	1
74	cross-sectoral collaboration with aligned goals for assessment and treatment.	4 (1–4)	4 (2–4)	3 (1–4)	1
80	listening to what the person says without drawing one's own conclusions prematurely.	4 (1–4)	4 (3–4)	3 (1–4)	1
81	understanding that the person has pain even when it is not visible.	4 (1–4)	4 (3–4)	3 (1–4)	1
82	ensuring that the person is taken seriously across both healthcare services and the job center. Healthcare professionals should not give patronising advice.	4 (1–4)	4 (3–4)	4 (1–4)	0
83	sharing knowledge between the job center, employer, and clinician about what best supports rehabilitation, so that everyone is working from the same understanding.	4 (2–4)	4 (2–4)	3 (2–4)	1
89	the municipality considering the value of requesting medical assessments, for example early after surgery. This may create the impression that rehabilitation is not progressing as expected and increase concern.	3 (1–4)	3 (1–4)	2 (1–4)	1
90	predictability/transparency in contact with the municipality. Clear agreements about the timing and content of calls, so that the person is not constantly required to account for how they are feeling and when they are ready to return to work.	3 (2–4)	4 (3–4)	3 (2–4)	1
94	bringing all information together—from hospital, general practitioner, specialist, dentist, physiotherapist, chiropractor, etc.—to create an overall overview of what the person has been referred to and has already tried.	3 (1–4)	4 (3–4)	3 (1–4)	1
102	vocational guidance with a broader perspective on employment and education.	3 (2–4)	3 (2–4)	3 (2–4)	0
110	initiating the necessary treatment as quickly as possible while also starting the intervention for the person on sick leave, so that the process does not become passive and waiting-oriented, but instead active on all fronts.	4 (1–4)	4 (4–4)	3.5 (1–4)	0.5
114	possibility of temporary provision of assistive devices.	3 (2–4)	4 (3–4)	3 (2–3)	1

118	help for the person on sick leave to navigate the systems, understand potential treatments, and identify support options.	3 (2–4)	4 (3–4)	3 (2–4)	1
119	early contact with the job center (or whatever it will be called in the future) and convening a meeting with the employer regarding work retention (work accommodations, etc.).	3 (1–4)	3 (1–4)	3 (2–4)	0
121	applying a long-term economic perspective on costs versus gains related to whether the patient remains on sick leave or returns to work—all factors included.	3 (1–4)	4 (1–4)	2 (1–4)	2

Cluster 6: Individual Support

		**Median (range)**			
**No.**	**Statement**	**Total (n = 36)**	**People with lived experience (n = 11)**	**Professionals (n = 21)**	**Median difference**
9	destigmatisation, even when the condition is described merely as "wear and tear."	3 (1–4)	4 (2–4)	3 (1–4)	1
13	equipping relatives with knowledge about persistent pain so that they are better able to understand and talk with the person with pain. This may make it easier, for example, for a family to organise daily life appropriately for all parties.	3 (1–4)	4 (2–4)	3 (1–4)	1
30	creating a sense of meaning for the individual in relation to pain, exercise, and work.	4 (2–4)	4 (2–4)	4 (3–4)	0
35	supporting the individual to see possibilities rather than limitations.	4 (2–4)	4 (3–4)	3 (2–4)	1
36	recognising the importance of involving significant others or relatives, as this may create greater understanding of the individual's challenges.	3 (1–4)	3 (3–4)	3 (1–4)	0
51	support for managing one's own situation throughout the rehabilitation process.	3.5 (2–4)	4 (3–4)	3 (2–4)	1
53	better understanding of the real challenge: everyone asks for systematics, standardisation, and plans, but people are different and so is pain; therefore, no single standard plan can be made. Individuality is what makes it difficult for the system.	3.5 (1–4)	4 (2–4)	3 (1–4)	1
54	focus on the person's own rehabilitation goals.	3 (1–4)	3 (3–4)	3 (1–4)	0
65	dialogue tools for advisers focusing on conversations about energy management.	3 (1–4)	3 (2–4)	2 (1–4)	1

66	increasing advisers' awareness that thoughts, worries, psychological strain, and life trauma may also be triggering/contributing factors and may therefore need to be included in the intervention plan.	4 (1–4)	4 (3–4)	4 (1–4)	0
75	the person experiencing that he/she is being heard.	4 (3–4)	4 (4–4)	4 (3–4)	0
76	understanding of their situation, that is, how pain affects their life, mood, functioning, thoughts, and other aspects.	4 (1–4)	4 (3–4)	3 (1–4)	1
97	screening from the outset for other problems (health-related or social) that may complicate the process, and focusing on these early.	3 (2–4)	4 (2–4)	3 (2–4)	1
100	parallel focus on positive aspects of life content.	4 (2–4)	4 (3–4)	4 (2–4)	0

Cluster 7: Comprehensive Assessment

		**Median (range)**			
**No.**	**Statement**	**Total (n = 36)**	**People with lived experience (n = 11)**	**Professionals (n = 21)**	**Median difference**
14	awareness among professionals that fear of movement may be a maintaining factor.	3 (1–4)	3 (1–4)	3 (2–4)	0
15	support from professionals to help the patient return to everyday life and work as early as possible.	4 (2–4)	4 (2–4)	4 (2–4)	0
18	assessment of the overall life situation (everyday life and work).	3 (1–4)	4 (2–4)	3 (1–4)	1
19	free or low-cost treatment/physiotherapy, as people on sick leave are often financially constrained.	3 (1–4)	4 (3–4)	3 (1–4)	1
24	assistive devices at home and possibly also at work.	3 (2–4)	4 (2–4)	2 (2–4)	2
29	a professional who dares to place physical demands and address mental barriers.	3.5 (1–4)	4 (1–4)	3 (1–4)	1
44	clarifying whether the person actually wants to return to the labour market/workplace. If "flex job" or "senior pension" has effectively been decided on early, that is often where the process ends.	3 (1–4)	3 (1–4)	3 (1–4)	0
50	reframing the problem with greater focus, from both patient and system, on functional limitations and how these can be improved. For years, both research and clinical practice have focused on pain as the central issue, but this has not moved things forward.	3 (2–4)	3 (2–4)	3 (2–4)	0

57	awareness that stress/depression as a secondary condition often develops at a point when treatment options have been exhausted.	3 (1–4)	4 (3–4)	3 (1–4)	1
60	coping programs within the job center involving both a physiotherapist and a psychologist.	3 (1–4)	3 (1–4)	3 (1–4)	0
64	focus on whether the individual has developed the ability to set limits, or whether support/compensation is needed through others acting as a brake—and advisers and job consultants also becoming aware of their role in this.	3 (1–4)	4 (3–4)	3 (1–4)	1
99	medical assessment that can reassure the patient and ensure that they are not afraid or worried.	3 (1–4)	4 (2–4)	3 (1–4)	1
120	if there is no improvement after 8–12 weeks, referral should be made for further assessment at a spine center, in accordance with the right to timely assessment.	3 (1–4)	4 (3–4)	3 (1–4)	1

Supplementary Table SIII. Adjustments to the concept map following the validation workshop

Change type	Description
Cluster renaming	Cluster 1 was renamed *Support for Self-management*; Cluster 2 was renamed *Physical Training and Everyday Movement*; Preliminary Cluster 6 was renamed *Extended Assessment and Support for Treatment*
Cluster restructuring	The preliminary Cluster 4 was dissolved, and its statements were moved to Clusters 2 and 3.
Cluster reduction	The concept map was reduced from 7 to 6 clusters
Statement relocation	17 statements were reallocated as follows in the final cluster solution:<br>• To Cluster 1 (Support for Self-management): 51, 76, 100<br>• To Cluster 2 (Physical Training and Everyday Movement): 39, 45, 95, 113<br>• To Cluster 3 (The Workplace): 20, 72, 79, 96, 103<br>• To Cluster 4 (Interdisciplinary Coordinated Intervention): 24, 53, 60, 97<br>• To Cluster 6 (Extended Assessment and Support for Treatment): 86
